# Draft Genome Sequence of Blood-Origin Streptococcus canis Strain FU149, Isolated from a Dog with Necrotizing Soft Tissue Infection

**DOI:** 10.1128/MRA.00737-20

**Published:** 2020-08-27

**Authors:** Yasuto Fukushima, Yoshiteru Murata, Yukie Katayama, Yuzo Tsuyuki, Haruno Yoshida, Tetsuya Mizutani, Takashi Takahashi

**Affiliations:** a Laboratory of Infectious Diseases, Graduate School of Infection Control Sciences, Ōmura Satoshi Memorial Institute, Kitasato University, Minato, Tokyo, Japan; b Murata Animal Hospital, Mobara, Chiba, Japan; c Research and Education Center for Prevention of Global Infectious Diseases of Animals, Tokyo University of Agriculture and Technology, Fuchu, Tokyo, Japan; d Division of Clinical Laboratory, Sanritsu Zelkova Veterinary Laboratory, Koto, Tokyo, Japan; University of Maryland School of Medicine

## Abstract

The draft genome sequence of the blood-origin Streptococcus canis strain FU149, isolated from a dog with a necrotizing soft tissue infection in Japan, is reported. The genome size was 2.108 Mbp, with a G+C content of 39.5%. Sequences unmapped to the reference genome sequence of NCTC 12191^T^ (GenBank accession number LR134293) were characterized.

## ANNOUNCEMENT

Streptococcus canis, which was first proposed in 1986 (1), is characterized as having beta-hemolysis activity and as Lancefield carbohydrate antigen group G. This bacterium can cause mild to severe infections in humans and animals (2–5). Here, we report the draft genome sequence of an S. canis strain isolated from the blood of a miniature sausage dog (male, 13 years old, born in Hiroshima City, Japan) with a necrotizing soft tissue infection.

The blood-origin strain FU149 was isolated using the blood culture system VersaTrek (Kohjin-Bio, Japan) (6). Isolates were inoculated onto sheep blood agar plates and incubated in 5% CO_2_ at 35°C for 24 h (3). Colonies with a gray-white smooth appearance (indicating beta-hemolysis) were further grown overnight in Todd-Hewitt broth supplemented with yeast extract. We picked up a single colony and extracted its genomic DNA using a DNeasy blood and tissue kit (Qiagen, Germany) after pretreating it with lysozyme and proteinase K. DNA samples were stored at −70 to −80°C until used. The DNA sequencing library was generated using a Nextera XT DNA sample prep kit (Illumina, USA). Sequencing was performed on an Illumina MiSeq benchtop sequencer. Paired-end runs were performed with read lengths of 2 × 75 bp.

Sequencing yielded 8,071,438 reads (604,909,372 bases). Reads were trimmed based on the quality trimming tool in CLC Genomics Workbench (v.12.0) with default parameters. *De novo* assembly was performed using CLC Genomics Workbench with modified parameters, in which the minimum contig length setting was changed from 200 to 500 bp. Draft genome sequences were annotated using the DDBJ Fast Annotation and Submission Tool (DFAST; https://dfast.nig.ac.jp) (7). Assembly metrics and annotated features included the genome size (2,108,133 bp), number of contigs (74), average coverage (284×), *N*_50_ value (73,653 bp), numbers of coding DNA sequences (CDSs; 2,049), tRNAs (18), rRNAs (2), and clustered regularly interspaced short palindromic repeats (2), G+C content (39.5%), and coding ratio (84.6%).

Mapping FU149 reads to the reference genome sequence of S. canis NCTC 12191^T^ (GenBank accession number LR134293) was performed using the reference tool with default parameters in the CLC Genomics Workbench. We attempted *de novo* assembly using the remaining unmapped reads (1,005,451 reads) and yielded 35 contigs. These contigs were uploaded into Web-based applications PathogenFinder v.1.1 (https://cge.cbs.dtu.dk/services/PathogenFinder/; Center for Genomic Epidemiology) (8) and DFAST to identify any pathogenic gene families that were not present in NCTC 12191^T^. Seven CDSs (position 1164 to 207 bp) were recognized by both applications (Table 1). These CDSs were found to encode DNA integration (contig number 49) and phage/phage-associated proteins (contig numbers 32 and 29) identical to those in pathogenic streptococci (Streptococcus dysgalactiae subsp. *equisimilis* and Streptococcus pyogenes), suggesting genetic transmission between animal-derived S. canis and other pathogenic streptococci.

**TABLE 1:** Characteristics of *Streptococcus canis* strain FU149^*a*^ pathogenic gene families that were not present in NCTC 12191^T^,^*b*^ identical to other pathogenic streptococci

Other pathogenic streptococci^*c*^	GenBank accession no.	Protein accession no.	Data from PathogenFinder v1.1^*d*^					Data from DFAST^*e*^			
			Product	Contig no.	Nucleotide start	Nucleotide end	Size (bp)	Product	Contig no.	Nucleotide start	Nucleotide end
Streptococcus dysgalactiae subsp. *equisimilis* GGS_124	AP010935.1	BAH82621	DNA integration/recombination/inversion protein	49	19755	20918	1,164	Site-specific integrase	49	19755	20918
Streptococcus pyogenes MGAS10394	CP000003.1	AAT86183	Unknown phage protein	32	6937	7824	888	Hypothetical protein	32	6937	7824
Streptococcus pyogenes MGAS5005	CP000017.2	AAZ52060	Phage transcriptional activator	32	534	950	417	Hypothetical protein	32	534	950
Streptococcus pyogenes MGAS5005	CP000017.2	AAZ52050	Phage protein	32	8203	8481	279	Hypothetical protein	32	8203	8481
Streptococcus pyogenes MGAS6180	CP000056.2	AAX72951	Hypothetical protein	29	2524	2751	228	Hypothetical protein	29	2524	2751
Streptococcus pyogenes MGAS5005	CP000017.2	AAZ52045	Phage protein	32	10380	10613	234	Hypothetical protein	32	10380	10613
Streptococcus pyogenes MGAS9429	CP000259.1	ABF31726	Phage protein	29	57517	57723	207	Hypothetical protein	29	57517	57723
Streptococcus pyogenes MGAS5005	CP000017.2	AAZ52055	Hypothetical protein	32	5814	5930	117	Not detected			

a Accession number BLRR01000000.

b GenBank accession number LR134293.1.

c We found the data regarding the other pathogenic streptococci, accession numbers, and protein numbers using the PathogenFinder v 1.1.

d The PathogenFinder v.1.1 (https://cge.cbs.dtu.dk/services/PathogenFinder/; Center for Genomic Epidemiology) was applied to obtain an overview of the genomic pathogenic gene families.

e The DDBJ Fast Annotation and Submission Tool (DFAST; https://dfast.nig.ac.jp/) was used to confirm the products (coding DNA sequences), contig numbers, and nucleotide starts/ends obtained using PathogenFinder.

We also determined the S. canis M-like protein (SCM) allele (9), sequence type (ST) by MLST v.2.0 (https://cge.cbs.dtu.dk/services/MLST/; Center for Genomic Epidemiology) (10), and antimicrobial resistance (AMR) genotype by ResFinder v.3.2 (https://cge.cbs.dtu.dk/services/ResFinder/; Center for Genomic Epidemiology) (11) using contigs. This strain showed a previously described pathogenic ST9 harboring SCM allele 1 without AMR genes (12).

### Data availability.

The draft genome sequence of S. canis has been deposited in DDBJ/EMBL/GenBank under accession number BLRR00000000.1, with SRA accession number DRR221832.
